# Announcement: International Biometals Symposium

**DOI:** 10.1155/MBD.1996.113

**Published:** 1996


					INTERNATIONAL BIOMETALS SYMPOSIUM
The University of Calgary
August 10 14, 1997
ORGANIZING COMMITTEE
Edward J. Laishley, The University of Calgary
GLinther Winkelmann, Universit&t T0bingen
Larry L. Barton, University of New Mexico
SCIENTIFIC COMMITTEE
Robert Huber, Nobel Laureate, Max-Planck Institut for Biochemie, Planegg, Martinsried
Thressa Stadtman, NIH, Bethesda
Prem Ponka, Jewish General Hospital, Montreal
Volkmar Braun, Universitt Tbingen
Simon Silver, University of Illinois, Chicago
Alan Baker, University of Sheffield
Jean Le Gall, University of Georgia
Joseph Coleman, Yale University
Wilfried Rauser, University of Guelph
Kenneth Raymond, University of California, Berkeley
Monica Nordberg, Karolinska Institutet, Stockholm
Dennis Winge, University of Utah
SCIENTIFIC PROGRAMME
The objective of the Congress is to bring together scientists to discuss topics of wide current
interest. Throughout, we would like to promote a forum for interdisciplinary participation and
exchange of ideas for research and interpretation of results
INTEREST GROUPS
The conference is intended to be of interest to Medical Scientists, Biochemists, Toxicologists,
Microbiologists, Environmental Scientists, Physiologists, Chemists, Geneticists, Molecular
Biologists, Cell Biologists
SITE AND DATE
The International Biometals Symposium will be held at The University of Calgary, August 10-14,
1997
SECOND CIRCULAR
The second circular will be published in late 1996 and will contain further information about
registration, the scicntific program, travel arrangements, accommodations and social events. The
second circularwill be sent only to those who ask for it by writing to:
International Biometals Symposium
The University of Calgary, Conference Management Services, Attn: Margaret-Anne Stroh
Olympic Volunteer Centre, 1833 Crowchild Trail N W, Calgary, Alberta T2M 4S7, Canada
Telephone: (403) 220-6229, Fax: (403) 284-4184
SCOPE OF THE CONFERENCE
The following themes are suggested as examples to show the intended breadth of the conference
Iron metabolism and overload in humans
Photoremediation studies
Biochemistry of metals
Metal centres in enzymes
Metals in transcription and translation
Metal complexes of biological interest
Transport and reduction of metal ions
Toxicitics due to metals
Special roles of transition elements
Metals and biotechnology
113

				

